# The Effect of Silicon on Osmotic and Drought Stress Tolerance in Wheat Landraces

**DOI:** 10.3390/plants10040814

**Published:** 2021-04-20

**Authors:** Sarah J. Thorne, Susan E. Hartley, Frans J. M. Maathuis

**Affiliations:** 1 Department of Biology, University of York, York YO10 5DD, UK; st945@york.ac.uk; 2 Department of Animal and Plant Sciences, University of Sheffield, Sheffield S10 2TN, UK; s.hartley@sheffield.ac.uk

**Keywords:** silicon fertiliser, wheat, osmotic stress, drought stress, landraces, genotypic variation

## Abstract

Drought stress reduces annual global wheat yields by 20%. Silicon (Si) fertilisation has been proposed to improve plant drought stress tolerance. However, it is currently unknown if and how Si affects different wheat landraces, especially with respect to their innate Si accumulation properties. In this study, significant and consistent differences in Si accumulation between landraces were identified, allowing for the classification of high Si accumulators and low Si accumulators. Landraces from the two accumulation groups were then used to investigate the effect of Si during osmotic and drought stress. Si was found to improve growth marginally in high Si accumulators during osmotic stress. However, no significant effect of Si on growth during drought stress was found. It was further found that osmotic stress decreased Si accumulation for all landraces whereas drought increased it. Overall, these results suggest that the beneficial effect of Si commonly reported in similar studies is not universal and that the application of Si fertiliser as a solution to agricultural drought stress requires detailed understanding of genotype-specific responses to Si.

## 1. Introduction

Wheat is the primary source of calories for 30% of the world population [1], with over 765 million tonnes of wheat produced globally in 2019 [2]. However, drought causes average global yield losses of ≈20% for wheat [3]. Anthropogenic climate change is predicted to induce changes in global precipitation patterns, with droughts likely to become more frequent in some areas, further exacerbating yield losses [4,5]. The impacts of abiotic stresses such as drought may be exacerbated by the focus of crop domestication on optimising yields at the expense of reduced stress tolerance and genetic diversity [6]. Current mitigation strategies include crop irrigation, but this often results in soil salinisation [7], and breeding for increased drought tolerance, particularly by using the genetic diversity of landraces to breed cultivars with improved drought tolerance [8]. However, breeding approaches are slow, labour intensive and complicated by genotype-environment interactions [9].

Silicon (Si) is the second most abundant element in the earth’s crust [10]. Although not considered an essential element in plants, Si accumulation has been linked to improved growth, especially in plants under stress [11]. However, plants only absorb Si as silicic acid, which is often limited in the soil [12]. In grasses, silicic acid is transported through the roots by the action of two transporters: Lsi1 and Lsi2 [13]. While most silicic acid is transported to the shoots, some is deposited in the roots, predominantly in the tangential and radial walls of endo- and exodermal tissues [14,15]. The majority of absorbed Si passes through the transpiration stream to the shoots [16]. In rice, the Si transporters Lsi2, Lsi3 and Lsi6 are involved in unloading silicic acid out from the xylem into the shoot [17]. High levels of silicic acid in the shoot cause its autopolymerisation into silica [18]. Deposited silica can be found in the form of phytoliths, silicified spines and surface structures, which occur in a range of shoot tissues [19,20]. Silica also accumulates in or beneath the cuticle layer of the cell wall in epidermal cell layers and tissues that surround the vasculature [18,21,22,23].

Si fertilisation may improve drought tolerance, although the exact underpinning mechanisms are unknown [24]. Drought stress induces oxidative damage [25], and Si fertilisation has been shown to reduce oxidative damage, notably by increasing antioxidative enzyme activity [26,27,28]. Additionally, Si fertilisation can improve water use efficiency during drought stress [28,29,30], for example, via an increase in stomatal conductance, which in turn improves the photosynthetic rate [31,32,33]. In contrast, other studies showed improved water use efficiency linked to lower transpiration, which may occur via reduction in cuticular water conductance [34,35].

Most studies on wheat report that Si increases growth and yield under drought stress [28,36,37,38], although there are others reporting no significant effect either in wheat [39] or in other species [40,41]. The inconsistent nature of the observed Si effects may reflect variation in plant species and genotype, as has been found for understanding the Si effect on herbivory tolerance [20,42,43]. For example, genotypic variability in Si accumulation is likely to affect the Si response, and while many studies assessed the effect of Si on cultivars that differ in drought tolerance [44,45,46], there is a clear lack of studies examining the effect of Si on drought tolerance in a larger range of genotypes, particularly those that vary in Si accumulation.

Although there is increasing interest in the use of local landraces in crop breeding programs to improve stress tolerance [8,47], to date, only a few studies have used landraces to investigate the effect of Si on osmotic and drought stress in wheat [48,49]. In this study, whether there are consistent differences in Si accumulation between wheat landraces was assessed. Whether there is a different effect of Si on osmotic and drought stress in landraces with relatively high Si content compared to those with lower Si content was then examined. It was hypothesised that the impact of Si on stress tolerance in landraces depends on their capacity to accumulate Si.

## 2. Materials and Methods

### 2.1. Plant Material, Experimental Design and Growth Conditions

#### 2.1.1. Wheat Diversity Panel

A diversity panel of 98 *Triticum aestivum* landraces, which is part of the 350 landraces YoGI biodiversity panel (Harper, unpublished data), was used. The panel was formed using material from the following collections: The International Maize and Wheat Improvement Center (CIMMYT), Mexico; Crop Research Institute, Prague; and John Innes Germplasm Resource Unit, the Biotechnology and Biological Sciences Research Council Designing Future Wheat programme. A full list of landraces is available in Table S1 in the Supplementary Materials.

#### 2.1.2. Growth of Wheat in Compost

Seeds for each *T. aestivum* landrace were planted in 500 mL pots filled with F2 + S compost (Levington) and placed in controlled glasshouse (15–20 °C, 16:8 hr light:dark). Prior to seed planting, pots filled with compost were treated with Calypso insecticide (Bayer) according to the manufacturer’s instructions. One week after germination, the seedlings were thinned to 2 plants per pot. The plants were grown in the glasshouse for 7 weeks and watered as required with tap water. The plants were cultivated with or without Si fertilisation. For plants grown with Si, the plants received 100 mL 1.5 mM sodium metasilicate (Sigma-Aldrich, St. Louis, MO, USA) twice weekly, starting one week after germination and continuing for 42 days when the shoots were sampled. All experiments consisted of four temporally separate replicates, with at least two weeks between experiments, apart from the screen using the diversity panel, which consisted of three replicates. Each replicate consisted of one plant per landrace per treatment. At harvest, the shoot fresh weight was recorded, the plants were oven-dried at 70 °C for 72 h to obtain shoot dry weights, and shoot Si concentration was measured as described below.

#### 2.1.3. Growth of Wheat in Hydroponics

The seeds of landraces were germinated in the glasshouse in sand for 10–11 days, and then, the seedlings were transferred to 9 L hydroponics boxes, filled with 1/2 strength Hoagland’s solution supplemented with Si as indicated. The pH was adjusted to 5.6–6.0 using 1 M HCl or 0.1 M KOH. For Si fertilisation, sodium metasilicate (Sigma-Aldrich, St. Louis, MO, USA) was added to achieve final Si concentrations of 0.2, 0.9 or 1.8 mM. Across all levels of Si availability, sodium chloride was used to balance sodium levels. The nutrient solution was changed every 3–4 days. The hydroponics boxes were aerated throughout the experiment. The plants were grown under controlled glasshouse conditions (15–20 °C, 16:8 hr light:dark) and sampled 6 weeks after germination. At the end of the experiment, the roots were washed in deionised water and the shoot and root fresh weights were recorded. The plants were oven-dried at 70°C for 72 h to obtain dry weights. The shoot and root Si concentrations were measured as described below.

#### 2.1.4. Imposition of Stress Conditions

To impose osmotic stress, the hydroponically grown plants were exposed to 8% (*w/v*) polyethylene glycol-(PEG)6000 (Sigma-Aldrich, St. Louis, MO, USA), which was calculated to have an osmotic pressure of −0.12 MPa [50]. The plants were treated for 4 weeks, and the experiment was composed of four replicates, with a minimum of two weeks between replicates. 

Drought stress was applied to the soil-grown plants by withholding watering until 40% field capacity (FC) was achieved. All plants were then watered as required to maintain the soil moisture at either control (100% FC) or drought (40% FC) levels. The soil moisture content was checked using a soil moisture probe (ML3 ThetaProbe Soil Moisture Sensor, delta-T). The plants were harvested 6 weeks after sowing. Four replicates were performed, with a minimum of two weeks between replicates and two plants per landrace per treatment per replicate.

### 2.2. Silicon Measurements

Shoot and root Si concentrations were measured by portable X-ray fluorescence spectroscopy (P-XRF) using the method described in Reidinger et al. [51]. The dried leaf material was ball-milled (Retsch MM400 Mixer mill, Haan, Germany), and the ground material was pressed at 10 tons into pellets using a manual hydraulic press with a 13 mm die (Specac, Orpington, UK). Si analysis (% Si dry weight) was performed using a commercial P-XRF instrument (Nitron XL3t900 GOLDD analyser: Thermo Scientific Winchester, UK) held in a test stand (SmartStand, Thermo Scientific, Winchester, UK). The P-XRF machine was calibrated using Si-spiked synthetic methyl cellulose (Sigma-Aldrich, product no. 274429) and validated using Certified Reference Materials of NCS DC73349 ‘Bush branches and leaves’ obtained from the China National Analysis Center for Iron and Steel. To avoid signal loss by air absorption, the analyses were performed under a helium atmosphere [50]. A reading of each side of the pellet was taken approximately one hour apart to account for u-drift in the instrument (i.e., variation in readings between consecutive runs using identical parameters [52]). The two readings were averaged to estimate the Si concentration (%). 

### 2.3. Statistical Analysis

All statistical analyses were performed using R software [53] (version 3.6.1). Three-way analysis of variance (ANOVA) was used to test the effect of Si fertilisation, stress treatment, and landrace or accumulation type on Si concentration, dry weight and stress tolerance. Stress tolerance in non-fertilised and Si fertilised conditions was calculated as the ratio of growth of stressed plants compared to control plants (Stress/Control and Stress + Si/Control + Si, respectively). In all ANOVAs, temporal replicate was included as a factor to account for variation caused by plants that were grown at different times. Data normality was checked using Shapiro tests, and homogeneity of variance was tested using Levene’s tests. Dry weights and stress tolerance were log transformed, and Si concentration was logit transformed to satisfy the test assumptions. Post hoc Tukey’s HSD tests were used when significant interaction terms were present.

## 3. Results

### 3.1. Variation in Si Concentration in a Wheat Diversity Panel and Selection of High and Low Accumulators

**Figure 1:**
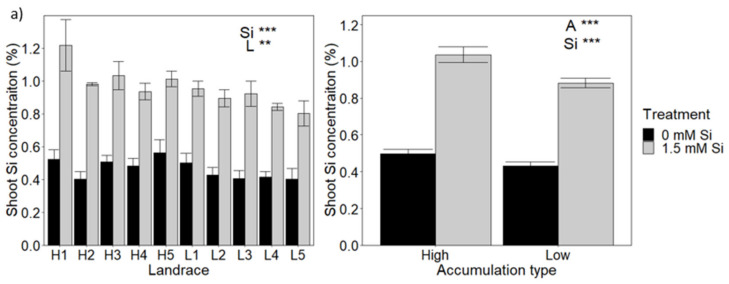

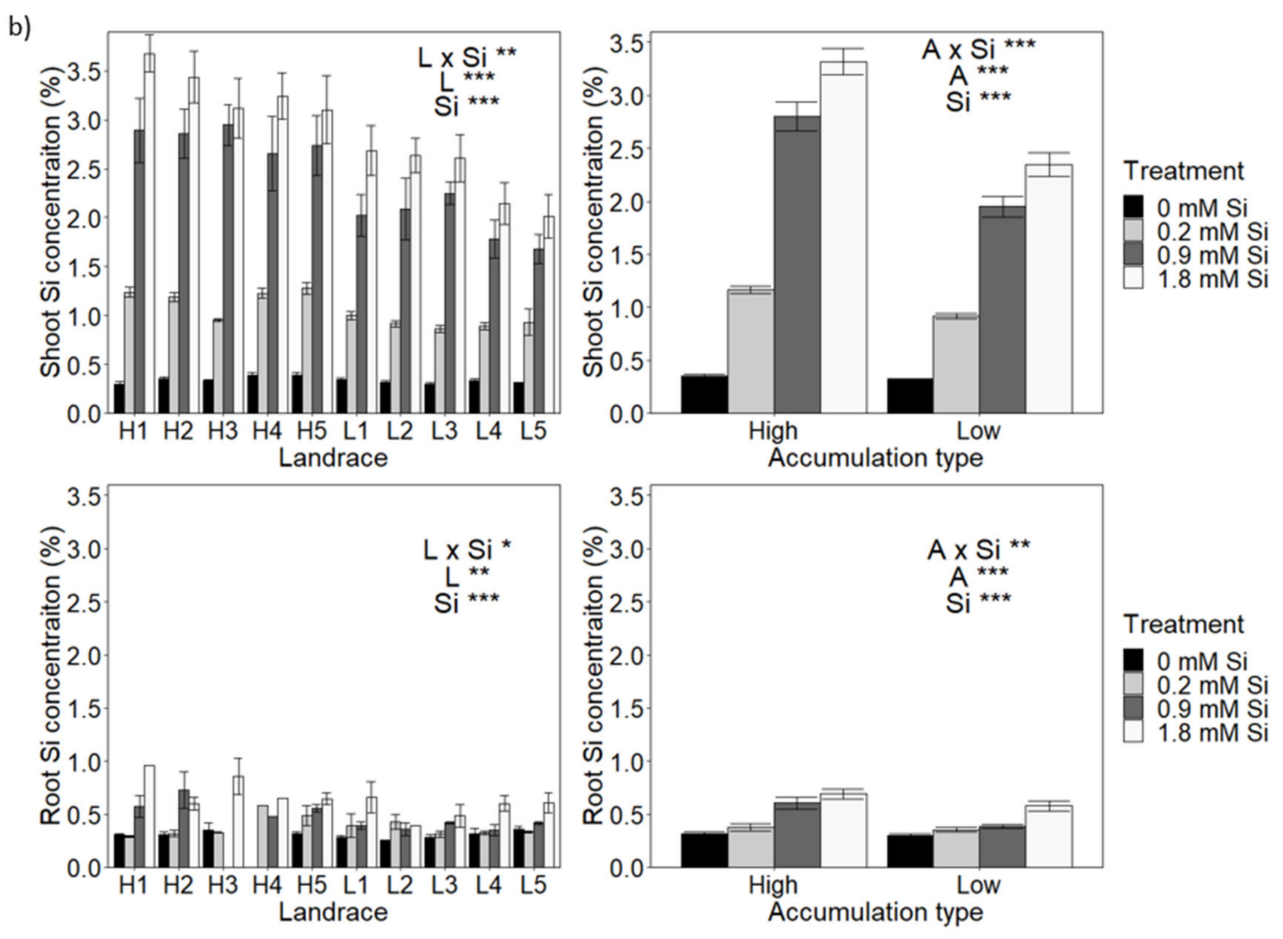
Variation in Si accumulation among selected wheat landraces. Ten wheat landraces were grown in (**a**) compost or (**b**) hydroponically with varying levels of Si fertilisation. Si level was measured for each landrace (left-hand panels). Average Si values, after classification into high and low Si accumulators, are shown in the right-hand panels. Statistically significant impacts and interactions, determined by 3-way ANOVA, are indicated in each panel, where *** *p* < 0.001, ** *p* < 0.01 and * *p* < 0.05. Mean values ± SE are shown. N = 3. L: Landrace, A: accumulation type, Si: level of Si fertilisation.

The 98 landraces were grown in compost and ranked according to their shoot Si levels for both the non-fertilised and Si fertilised conditions. The 10 highest and 10 lowest scoring landraces were then cultivated in hydroponics to control the Si supply more accurately and to assess whether ranking was consistent across growing systems and the amount of Si supplied to the growth medium. These combined analyses allowed for identification of five landraces that consistently showed relatively high and five landraces that consistently showed relatively low shoot Si accumulation for compost grown plants (Figure 1a) and hydroponically grown plants (Figure 1b). Furthermore, the plants from hydroponics showed that the shoot accumulation pattern was reproduced in root tissue (Figure 1b). Based on these data, a ‘high’ or ‘low’ Si accumulation type was assigned to each of the 10 landraces.

When cultivated in compost, shoot Si concentration in the selected 10 landraces was significantly affected by both landrace and Si fertilisation, but no interaction was found between these variables (Figure 1a; Table S2 in the Supplementary Materials). When grown with Si, the average Si concentration for the five high Si landraces was 1.04% compared to 0.88% for the five low Si landraces, a small but significant difference between accumulation types (Figure 1a; Table S2).

For hydroponically grown plants, Si fertilisation, landrace and the interaction between these factors, all had significant effects on shoot as well as root Si concentrations (Figure 1b; Table S2). The same pattern of significant effects on shoot and root Si contents were found for accumulation type (Figure 1b; Table S2). Thus, high accumulators not only accumulate more Si but also are more responsive to Si addition. For example, high accumulators showed bigger increases in Si uptake in response to 0.9 mM Si than low accumulators (Figure 1b). Shoot Si concentrations were higher in hydroponic-grown plants than in compost-grown ones, and the difference between high and low accumulators was greater, with an average of 3.24% Si for high accumulators grown in 1.8 mM Si compared to 2.35% Si for low accumulators.

### 3.2. The Effect of Accumulation Type and Si Supply on Plant Growth during Osmotic Stress

**Figure 2:**
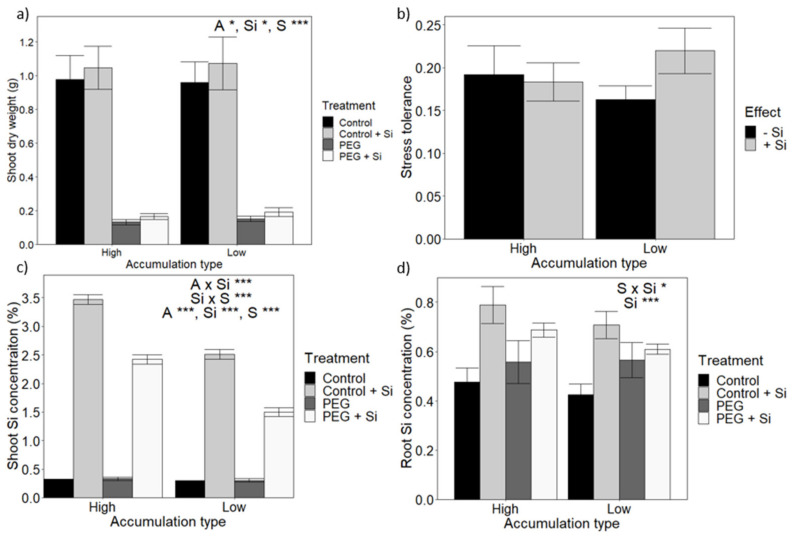
The impact of Si fertilisation on wheat osmotic stress tolerance according to Si accumulation type. (**a**) Shoot dry weight (DW). (**b**) Growth data were normalised to express ‘Stress tolerance’, defined as the ratio between shoot growth under osmotic stress compared to the control. (**c**) Shoot Si concentration. (**d**) Root Si concentration. Statistically significant impacts and interactions, determined by 3-way ANOVA, are indicated in each panel, where *** *p* < 0.001, ** *p* < 0.01 and * *p* < 0.05. Mean values ± SE are shown. N = 4. L: Landrace, A: accumulation type, Si: level of Si fertilisation, S: osmotic stress treatment.

Osmotic stress, Si fertilisation and accumulation type significantly affected shoot dry weight (Table S3). Si improved shoot biomass when plants were exposed to PEG for the high accumulation type only, and the effect of Si fertilisation was relatively small (Figure 2a). 

To facilitate comparisons between high and low Si accumulators, growth data were normalised and expressed as ‘Stress tolerance’, defined as the ratio of growth during stress compared to growth in the control. The stress tolerance was calculated separately for –Si and +Si plants. Neither Si fertilisation nor Si accumulation type significantly affected osmotic stress tolerance (Figure 2b). 

As can be seen in Figure 2c,d, during osmotic stress, the high and low Si accumulation traits were preserved, with significantly higher shoot and root Si levels for the high Si accumulators. Interestingly, for plants that were Si fertilised, an imposition of osmotic stress itself led to a large reduction in shoot, but not root, Si concentration relative to the results for the non-stressed plants.

Overall, no impact of Si on stress tolerance was observed in either the high or low Si accumulators. This lack of response may be due to the fact that Si impact is insignificant in all of the lines or because the positive Si effect of one line is cancelled by the negative response of another. However, Figure S1 shows that, across all the landraces, there was no significant effect of Si on stress tolerance in any of the landraces, ruling out the latter explanation.

### 3.3. The Effect of Accumulation Type and Si Supply on Growth during Drought Stress

Osmotic stress applied using chemical agents such as PEG is frequently applied to plants to mimic physiological drought. Such hydroponics-based assays have the advantage of exposing plants to a better controlled and less complex growth substrate and allows access to roots. However, genuine drought stress, i.e., water deficit, better simulates real field conditions. Furthermore, responses to osmotic and drought stress can be very different [54,55]. This study was therefore repeated using compost-grown wheat where water deficits could be applied. 

**Figure 3:**
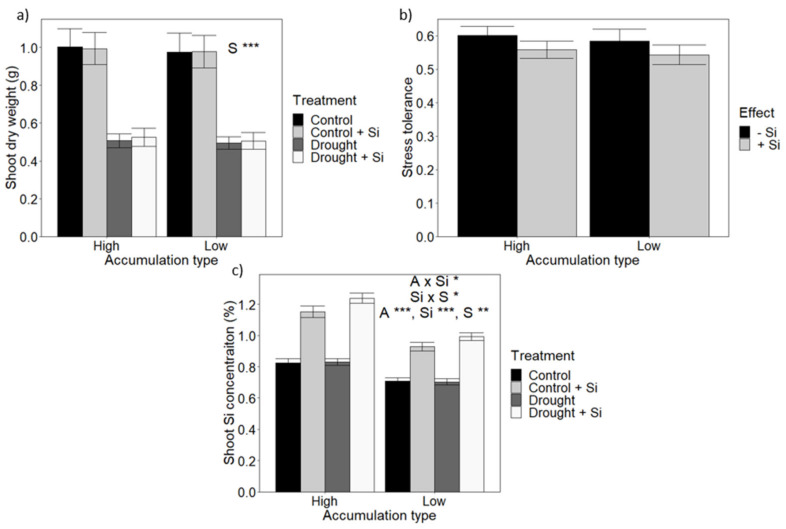
The impact of Si fertilisation on wheat drought stress tolerance. (**a**) Shoot dry weight (DW). (**b**) Growth data were normalised to express ‘Stress tolerance’, defined as the ratio between shoot growth under drought stress compared to the control. (**c**) Shoot Si concentration. Statistically significant impacts and interactions, determined by 3-way ANOVA, are indicated in each panel, where *** *p* < 0.001, ** *p* < 0.01 and * *p* < 0.05. Mean values ± SE are shown. N = 20. L: Landrace, A: accumulation type, Si: level of Si fertilisation, S: drought stress treatment.

Drought stress (i.e., 40% FC) significantly the lowered shoot dry weight by around 50% (Figure 3a). This reduction in biomass was the same for both high and low Si accumulators, and the addition of Si did not significantly alter the observed pattern (Table S4).

In addition to the effect of Si on absolute growth, the Si effect on stress tolerance was investigated. Stress tolerance (Figure 3b) was the same in high and low Si accumulators; Si fertilisation did not have a significant effect on tolerance to drought stress. 

Figure 3c shows that, for compost-grown plants in all four Si × drought treatments, the high accumulators had higher levels of shoot Si than the low accumulators. Furthermore and in contrast to the findings here for osmotic stress, when water availability was lowered, it caused an increase in shoot Si levels of Si fertilised plants (Figure 3c). This increase was larger for the high Si accumulators compared to the low Si accumulators. 

As with osmotic stress, the effect of Si on stress tolerance on each landrace was investigated to verify that the lack of Si response was not the result of positive effects in some landraces being cancelled by negative effects in other landraces. There was no significant effect of Si on stress tolerance for any individual landrace (Figure S2).

## 4. Discussion

Si fertilisation could be a cost-effective method to mitigate water stress in crops. However, reports on its efficacy vary widely and uncertainty regarding the underpinning mechanisms remain. An important question in this regard is whether and how variation in plant Si accumulation relates to Si impact on plant growth during water stress. Previous studies have investigated the differences in Si accumulation between genotypes [20,56,57] and the effect of Si accumulation on growth, including yield. For example, Merah et al. [48] found no significant correlation between Si content and grain yield among 10 durum wheat genotypes. However, to our knowledge, no studies have correlated the differences in Si accumulation with differences in stress tolerance. To address this question, whether there is a different effect of Si on osmotic and drought stress in landraces with high Si content compared to those with low Si content was examined. 

In spite of the significant differences in both shoot and root Si contents between the low and high accumulator groups (Figure 1) and with the exception of a small growth improvement for PEG-treated high accumulators (Figure 2), no difference in growth or stress tolerance was observed between accumulator types irrespective of treatment (Figure 2 and Figure 3). A similar conclusion is arrived at when investigating Si impact on individual landraces. Thus, these findings do not corroborate wheat studies that found that Si improves growth under osmotic [58] and drought stress [28,36,37,38]. Nevertheless, other studies on osmotic and drought-stressed wheat did not report a significant increase in shoot dry weight biomass [39,59]. Furthermore, a lack of response to Si has also been reported for many other crop species. For example, Ruppenthal et al. [60] reported that Si did not improve growth during drought in soybean, although Si did reduce membrane damage and increased peroxidase activity. In the case of barley, osmotic stress led to a rise in tissue Si but it did not alter biomass [40,41], and a similar result was reported in tall fescue [34].

These disparities may be (partly) due to methodological aspects; for instance, many studies used Na or K silicate as the Si treatment but failed to correct for cation concentrations in the control treatment. Hence, it is likely that the observed Si response is in fact due to extra Na or K fertilisation. Interpretational divergence is another potential source of confusion; many studies report a positive impact of Si on tolerance to osmotic or drought stress, when in reality, the effects of Si are already obvious in control treatments and hence are not stress specific. 

Genotype-specific Si responses could be another important factor; Hu et al. [61] found that the positive effect of Si on growth in poinsettia under control conditions was cultivar dependent. In sugarcane, the effect of Si under water-deficit conditions varied among cultivars, with a significant positive effect on dry weight observed in only one out of four tested cultivars [62]. Similar, genotype-specific effects of Si under drought stress have also been found when investigating 12 sunflower cultivars [63]. In wheat, Sapre and Vakharia [64] found variation both in the physiological response to osmotic stress and in Si accumulation among 10 wheat cultivars. 

Clearly, there is great, genotype-dependent variability in the Si effects, and this could go some way in explaining the lack of responses to Si within an accumulation type if positive and negative changes in stress tolerance for individual landraces tend to cancel each other. This possibility was tested by examining the responses of individual landraces to Si: the impact of Si fertilisation on stress tolerance did vary between landraces from around 25 to +40% during osmotic stress (Figure S1) and from around −30 to +10% during drought (Figure S2), but none of these changes was significant. 

Since Si fertilization did not change the stress tolerance, the hypothesis that Si correlates with tissue Si contents could not be properly tested. However, interesting interactions between stress and tissue Si content were found; during osmotic stress, the levels of Si in shoots were greatly reduced, whereas after exposure to drought, the opposite was observed, i.e., Si content increased. These effects were observed consistently across most landraces. Decreased Si accumulation in response to osmotic stress imposed using PEG has been previously reported [41,60,61,65]. Additionally, studies in both wheat [28,66] and other species have reported decreased Si accumulation during drought stress [29,67,68]. Nevertheless, increased Si during drought, as found in this study, has also been reported in the literature [30,69]. It is not clear what, if any, the physiological relevance is of these processes and whether they are part of a mechanistic link between Si accumulation and stress response. The similarity in physiological responses to osmotic and drought stress would, perhaps naively, suggest parallel changes in the accumulation of Si in response to both conditions, though they may be species dependent.

## 5. Conclusions

In this study, it was shown that wheat landraces varied significantly and consistently in their Si accumulation, such that two groups, high Si accumulators and low Si accumulators, could be identified. Osmotic stress decreased the Si content in both accumulation types whereas drought increased it. However, no significant effect of Si on growth during drought stress was found, and Si was found to improve growth only slightly in high Si accumulators during osmotic stress. The failure reported here to observe clear positive effects of Si suggest that, for wheat, any mitigation of the impacts of water stress by Si is limited. Further research is required to establish whether modern cultivars exhibit a more positive response toward Si fertilization compared to the landraces used in this study.

## Data Availability

Not applicable.

## Supplementary Materials

The following are available online at https://www.mdpi.com/article/10.3390/plants10040814/s1, Figure S1: The impact of Si fertilisation on individual wheat landraces under osmotic stress, Figure S2: The impact of Si fertilisation on individual wheat landraces under drought stress, Table S1: Shoot Si concentrations for the 98 landrace diversity panel, Table S2: ANOVA for the effect of Si fertilisation, for landrace or Si accumulation type, and for their interactions on the tissue Si contents of plants grown in compost or hydroponics, Table S3: ANOVA for osmotic stress, Si fertilisation and Si accumulation type and for their interactions on shoot dry weight, Si concentration and stress tolerance in hydroponic-grown plants, Table S4: ANOVA for osmotic stress, Si fertilisation and Si accumulation type and for their interactions on shoot dry weight, Si concentration and stress tolerance in compost-grown plants.
