# Supplementary material for: The Effect of Silicon on Osmotic and Drought Stress Tolerance in Wheat Landraces
Source: Plants (Basel). 2021 Apr 20;10(4):814. doi: 10.3390/plants10040814 (PMC8074377; doi:10.3390/plants10040814)
Supplement: Supplementary file 1 [file plants-10-00814-s001.zip › plants-1177584-supplementary.pdf]

Table S1: Shoot Si concentrations for the 98 landrace diversity panel. Landraces in bold were used for subsequent experiments. Landraces highlighted in bold were used for drought and ostmotic stress experiments.

Landrace	Collection	Plant ID	Plant Name	Origin	Shoot Si Concentration (%) (Mean ± SE)			
					0 mM Si		1.5 mM Si	
YoGI_002	CIMMYT	BW 7112	RA SHIH PAI PÂ΄I	China	0.47	0.03	0.99	0.07
YoGI_003	CIMMYT	BW7227	ARTEMOVKA	Former Soviet Union	0.41	0.08	0.86	0.05
YoGI_006	CIMMYT	BW 15958	V763.153	Pakistan	0.49	0.12	1.00	0.07
**YoGI_011**	**CIMMYT**	**CWI 2166**	**K7155.41**	**Kenya**	**0.52**	**0.06**	**1.22**	**0.16**
YoGI_013	CIMMYT	CWI 2168	K6995.4A	Kenya	0.47	0.02	0.91	0.05
**YoGI_015**	**CIMMYT**	**CWI 3909**	**OUBAARD**	**South Africa**	**0.51**	**0.04**	**1.03**	**0.08**
YoGI_021	CIMMYT	CWI 6075	KOELZ W 9375:AE	India	0.48	0.07	0.97	0.03
YoGI_022	CIMMYT	CWI 6076	KOELZ W 9376:AE	India	0.46	0.06	1.10	0.07
YoGI_028	CIMMYT	CWI 7129	KOELZ W 11192:AE	India	0.51	0.08	1.00	0.08
YoGI_034	CIMMYT	CWI 9915	ROOI KLEINKORING	South Africa	0.47	0.05	0.94	0.08
YoGI_038	CIMMYT	CWI 12335	AUSTRAL	Argentina	0.52	0.02	1.01	0.02
YoGI_047	CIMMYT	CWI 13432	WHITE FIFE	Japan	0.43	0.05	0.85	0.10
YoGI_051	CIMMYT	CWI 13629	AMERICANO	Uruguay	0.49	0.08	0.79	0.01
YoGI_052	CIMMYT	CWI 13644	RINK	United States	0.48	0.04	0.88	0.03
YoGI_054	CIMMYT	CWI 13647	NEW ZEALAND	United States	0.54	0.04	1.13	0.08
YoGI_059	CIMMYT	CWI 15005	LAGEADINHO	Brazil	0.47	0.06	0.89	0.05
YoGI_064	CIMMYT	CWI 27304	TAIAN-KEN	Kenya	0.42	0.04	0.85	0.05
YoGI_067	CIMMYT	CWI 31262	KENYA	Australia	0.45	0.02	0.84	0.05
YoGI_075	CIMMYT	CWI 52382	OAX93.21.34	Mexico	0.45	0.07	0.98	0.05
YoGI_079	CIMMYT	CWI 53414	NOVOMICHURINK A X	Former Soviet Union	0.44	0.04	0.93	0.04
YoGI_081	CIMMYT	DW 4633	AKBUGDAY	Kyrgyzstan	0.57	0.11	0.89	0.09
YoGI_085	Watkins	1190014	Sarajevo 4	Yugoslavia	0.44	0.07	0.96	0.05
YoGI_088	Watkins	1190032	Dehak	India	0.44	0.05	0.82	0.04
YoGI_098	Watkins	1190045	Douchani	Syria	0.41	0.05	1.00	0.03
YoGI_103	Watkins	1190091	Rodi Garamseli	India	0.50	0.06	1.08	0.05
YoGI_104	Watkins	1190092	Desi	India	0.43	0.08	0.87	0.06
YoGI_110	Watkins	1190105	Hâtif de Saône	France	0.50	0.11	0.96	0.04
YoGI_114	Watkins	1190126	Dolatkhani (white)	India	0.42	0.05	0.82	0.02
YoGI_118	Watkins	1190141	China 15	China	0.44	0.02	0.87	0.07
YoGI_130	Watkins	1190181	Kujawianka Wieclawicka	Poland	0.40	0.07	0.88	0.08
YoGI_133	Watkins	1190190	Roux de Presles	France	0.46	0.06	0.98	0.05
YoGI_134	Watkins	1190191	Rouge des Ardennes	France	0.42	0.03	0.89	0.01

**YoGI_135**	**Watkins**	**1190195**	**Gahu (Nepali) or Kyo (Sikkimese)**	**India**	**0.40**	**0.04**	**0.98**	**0.01**
YoGI_142	Watkins	1190218	Sbei Noir	Tunisia	0.42	0.04	0.94	0.05
YoGI_143	Watkins	1190219	Alicante 1	Spain	0.55	0.05	1.10	0.08
YoGI_144	Watkins	1190223	Shan wheat	Burma	0.44	0.05	0.93	0.05
YoGI_152	Watkins	1190246	Soor Ghanum	India	0.59	0.06	1.12	0.08
YoGI_156	Watkins	1190273	Seville 17	Spain	0.40	0.06	0.82	0.04
YoGI_162	Watkins	1190299	Smyrna 6	Turkey	0.49	0.05	0.91	0.05
YoGI_164	Watkins	1190305	Sinai 1	Egypt	0.43	0.05	0.97	0.04
YoGI_168	Watkins	1190319	China 15	China	0.43	0.08	0.86	0.09
YoGI_169	Watkins	1190320	China 2	China	0.45	0.06	1.05	0.09
YoGI_170	Watkins	1190323	China 14	China	0.44	0.03	0.91	0.05
YoGI_171	Watkins	1190324	China 20	China	0.49	0.07	0.94	0.04
YoGI_172	Watkins	1190325	1190325	United Kingdom	0.49	0.05	0.91	0.05
YoGI_178	Watkins	1190336	Hungary 2	Hungary	0.43	0.02	1.00	0.02
YoGI_181	Watkins	1190349	Golema Franga	Bulgaria	0.44	0.09	0.92	0.02
YoGI_182	Watkins	1190352	Sarajevo 8	Yugoslavia	0.45	0.04	1.11	0.05
YoGI_186	Watkins	1190374	Sarakhs	Iran	0.47	0.03	1.00	0.13
YoGI_189	Watkins	1190396	Trigo Rietti	Portugal	0.40	0.04	0.89	0.08
YoGI_190	Watkins	1190397	Trigo Ribeiro	Portugal	0.47	0.03	0.92	0.03
YoGI_191	Watkins	1190398	Abu Fashi	Palestine	0.48	0.06	1.05	0.12
YoGI_193	Watkins	1190406	Desi	India	0.51	0.06	0.93	0.10
YoGI_196	Watkins	1190420	Dhania	India	0.43	0.03	0.87	0.08
YoGI_197	Watkins	1190433	Soor Ghanum	India	0.48	0.06	0.89	0.02
YoGI_198	Watkins	1190436	China Sh108	China	0.41	0.03	0.93	0.06
YoGI_202	Watkins	1190450	Miercurea Ciucului	Romania	0.42	0.04	0.70	0.04
YoGI_203	Watkins	1190451	Samanta 117	Romania	0.46	0.04	0.79	0.06
YoGI_207	Watkins	1190468	Afghanistan 46	Afghanistan	0.48	0.04	0.89	0.08
YoGI_210	Watkins	1190474	Afghanistan 109	Afghanistan	0.43	0.07	0.87	0.07
YoGI_215	Watkins	1190483	Surka Oscista	Poland	0.42	0.04	1.04	0.13
**YoGI_221**	**Watkins**	**1190521**	**Dandi**	**India**	**0.50**	**0.06**	**0.95**	**0.05**
**YoGI_227**	**Watkins**	**1190568**	**China 19**	**China**	**0.43**	**0.05**	**0.89**	**0.05**
**YoGI_231**	**Watkins**	**1190605**	**Karabash**	**Greece**	**0.41**	**0.05**	**0.92**	**0.08**
YoGI_235	Watkins	1190627	Persia 45	Iran	0.48	0.05	0.95	0.07
YoGI_237	Watkins	1190636	Native hard	Tunisia	0.45	0.04	0.82	0.06
YoGI_240	Watkins	1190645	Mundia	India	0.45	0.05	1.00	0.06
YoGI_242	Watkins	1190652	China Sh107	China	0.44	0.03	0.95	0.06
**YoGI_243**	**Watkins**	**1190662**	**Samanta 1252**	**Romania**	**0.42**	**0.03**	**0.84**	**0.02**
YoGI_249	Watkins	1190670	Zlotka Miczynskiego	Poland	0.42	0.03	0.83	0.04
YoGI_255	Watkins	1190685	Trigo duros	Spain	0.44	0.04	0.86	0.00
YoGI_257	Watkins	1190694	Lyallpur 8A	India	0.49	0.02	0.96	0.03
YoGI_259	Watkins	1190700	Kaifeng 323-9	China	0.44	0.05	0.89	0.09

YoGI_261	Watkins	1190705	Kooseh	Iran	0.44	0.06	0.85	0.02
YoGI_265	Watkins	1190729	Gandum-i-Jiruft	Iran	0.44	0.03	0.78	0.01
YoGI_270	Watkins	1190740	Siberia W94421	USSR	0.49	0.10	0.89	0.02
**YoGI_277**	**Watkins**	**1190751**	**Armavir**	**USSR**	**0.40**	**0.07**	**0.80**	**0.08**
YoGI_280	Watkins	1190755	Crimea W94465	USSR	0.42	0.04	0.88	0.06
YoGI_285	Watkins	1190772	Yenisei W43320	USSR	0.50	0.04	0.87	0.04
**YoGI_286**	**Watkins**	**1190777**	**Finland 3**	**Finland**	**0.48**	**0.05**	**0.94**	**0.05**
YoGI_287	Watkins	1190779	Tulun 458	USSR	0.44	0.07	0.83	0.02
YoGI_291	Watkins	1190784	Oberdan	Italy	0.45	0.03	0.87	0.02
YoGI_298	Watkins	1190810	Turkestan W84532	USSR	0.47	0.05	0.93	0.08
YoGI_299	Watkins	1190811	Algeria W7558	Tunisia	0.59	0.14	0.97	0.11
YoGI_313	Prague	01C0200519	Eritrospermum 5755	Uzbekistan	0.47	0.03	0.87	0.04
YoGI_320	Prague	01C0203773	Jade	Saudi Arabia	0.50	0.06	0.98	0.03
YoGI_324	Prague	01C0202172	MCB 192	Peru	0.50	0.02	1.03	0.08
YoGI_328	Prague	01C0201384	Nohoean	Norway	0.44	0.02	0.86	0.02
YoGI_329	Prague	01C0201385	Nora	Norway	0.44	0.06	0.88	0.05
**YoGI_330**	**Prague**	**01C0201531**	**Orchon**	**Mongolia**	**0.56**	**0.08**	**1.01**	**0.05**
YoGI_334	Prague	01C0202818	Suwon 222	Korea	0.50	0.02	0.95	0.07
YoGI_336	IBTI	Unknown	Apache USA	Unknown	0.47	0.03	1.14	0.07
YoGI_343	IBTI	GedifluxRL (id#40001)	Muck	Germany	0.47	0.03	0.91	0.04
YoGI_345	IBTI	GedifluxRL (id#39977)	STAMM-101	Austria	0.45	0.03	0.99	0.05
YoGI_348	IBTI	GedifluxRL (id#39779)	Vilmorin-27	France	0.46	0.04	0.94	0.02
YoGI_349	IBTI	GedifluxRL (id#40037)	Shamrock	United Kingdom	0.49	0.03	1.15	0.07
YoGI_350	IBTI	Unknown	Paragon control	Unknown	0.47	0.04	0.88	0.07

Table S2: ANOVA for the effect of Si fertilisation, and landrace or Si accumulation type, and their interactions on tissue Si contents of plants grown in compost or hydroponics. Data were used to select landraces that varied consistently in their Si accumulation. Replicate was included as a random effect.

		Compost; shoot Si level			Hydroponics; shoot Si level			Hydroponics; root Si level		
		Df	F	p	Df	F	p	Df	F	p
Landrace	Landrace	**9**	**3.16**	**0.006**	**9**	**18.02**	**< 0.001**	**9**	**3.34**	**0.002**
	Si fertilisation	**1**	**346.08**	**< 0.001**	**3**	**2193.13**	**< 0.001**	**3**	**49.64**	**< 0.001**
	Replicate	**2**	**4.14**	**0.024**	**3**	**53.16**	**< 0.001**	3	1.26	0.296
	Landrace x Si	9	0.54	0.838	**27**	**2.06**	**0.004**	**25**	**1.99**	**0.012**
Accumulation type	Accumulation type	**1**	**13.41**	**< 0.001**	**1**	**74.66**	**< 0.001**	**1**	**13.76**	**< 0.001**
	Si fertilisation	**1**	**327.50**	**< 0.001**	**3**	**1841.11**	**< 0.001**	**3**	**48.17**	**< 0.001**
	Replicate	**2**	**3.92**	**0.026**	**3**	**42.20**	**< 0.001**	3	1.15	0.332
	Accumulation x Si	1	0.04	0.837	**3**	**6.79**	**< 0.001**	**3**	**4.26**	**0.007**

Table S3: ANOVA for osmotic stress, Si fertilisation, and Si accumulation type, and their interactions on shoot dry weight, Si concentration, and stress tolerance in hydroponic-grown plants. Replicate was included as a random effect to account for variability between plants grown at different times.

	Shoot dry weight			Shoot Si concentration			Root Si concentration			Stress tolerance		
	Df	F	p	Df	F	p	Df	F	p	Df	F	p
Accumulation type	**1**	**4.15**	**0.043**	**1**	**68.74**	**< 0.001**	**1**	**2.20**	**0.140**	1	2.50	0.119
Si fertilisation	**1**	**6.51**	**0.012**	**1**	**3195.82**	**< 0.001**	**1**	**43.70**	**< 0.001**	1	0.334	0.565
Stress treatment	**1**	**623.96**	**< 0.001**	**1**	**48.01**	**< 0.001**	**1**	**0.55**	**0.459**			
Replicate	**3**	**64.43**	**< 0.001**	**3**	**16.14**	**< 0.001**	3	2.51	0.062	**3**	**17.78**	**< 0.001**
Accumulation x Si	1	0.42	0.518	**1**	**22.20**	**< 0.001**	1	0.10	0.752	1	0.40	0.530
Accumulation x Stress	1	0.003	0.954	1	1.10	0.296	1	0.00	0.994			
Si x Stress	1	0.845	0.359	**1**	**31.05**	**< 0.001**	**1**	**4.11**	**0.045**			
Accumulation x Si x Stress	1	0.187	0.666	1	0.93	0.337	1	0.99	0.322			

Table S4: ANOVA for drought stress, Si fertilisation, and Si accumulation type, and their interactions on shoot dry weight, Si concentration, and stress tolerance in compost-grown plants. Replicate was included as a random effect to account for variability between plants grown at different times.

	Shoot dry weight			Shoot Si concentration			Stress tolerance		
	Df	F	p	Df	F	P	Df	F	P
Accumulation type	1	0.18	0.673	**1**	**162.16**	**< 0.001**	1	0.81	0.370
Si fertilisation	1	0.02	0.899	**1**	**540.44**	**< 0.001**	1	0.21	0.651
Stress treatment	**1**	**165.75**	**< 0.001**	**1**	**8.36**	**0.004**			
Replicate	**3**	**80.95**	**< 0.001**	**3**	**95.52**	**< 0.001**	**3**	**8.56**	**< 0.001**
Accumulation x Si	1	0.01	0.923	**1**	**4.57**	**0.033**	1	0.01	0.922
Accumulation x Stress	1	0.03	0.873	1	0.33	0.565			
Si x Stress	1	0.27	0.603	**1**	**5.29**	**0.022**			
Accumulation x Si x Stress	1	0.16	0.690	1	0.02	0.877			

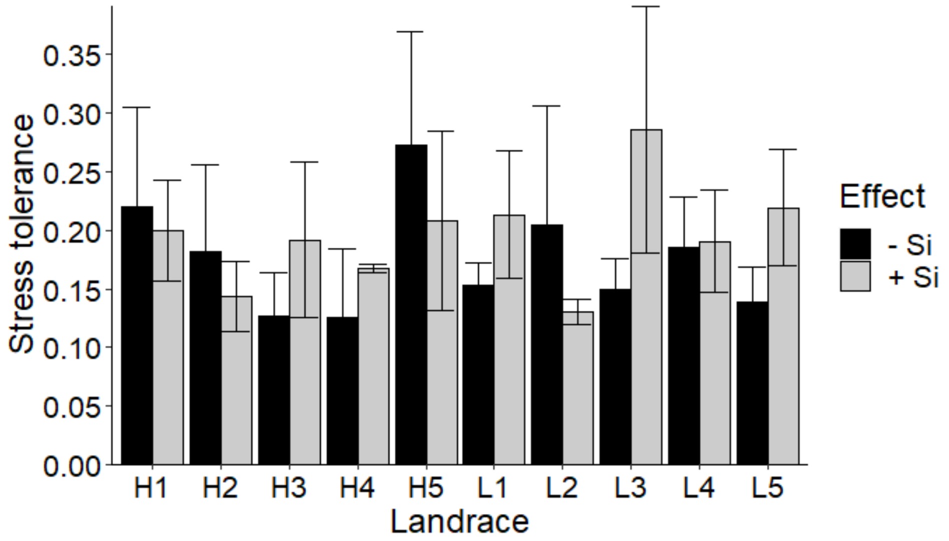

Figure S1: The impact of Si fertilisation on individual wheat landraces under osmotic stress. Stress tolerance was defined as shoot dry weight during osmotic stress divided by shoot dry weight in the control. Paired t-tests were used to compare the stress tolerance for – Si and + Si plants for each landrace. The effect of Si was not significant for any landrace (p > 0.05). Mean values ± SE are shown. N = 4.

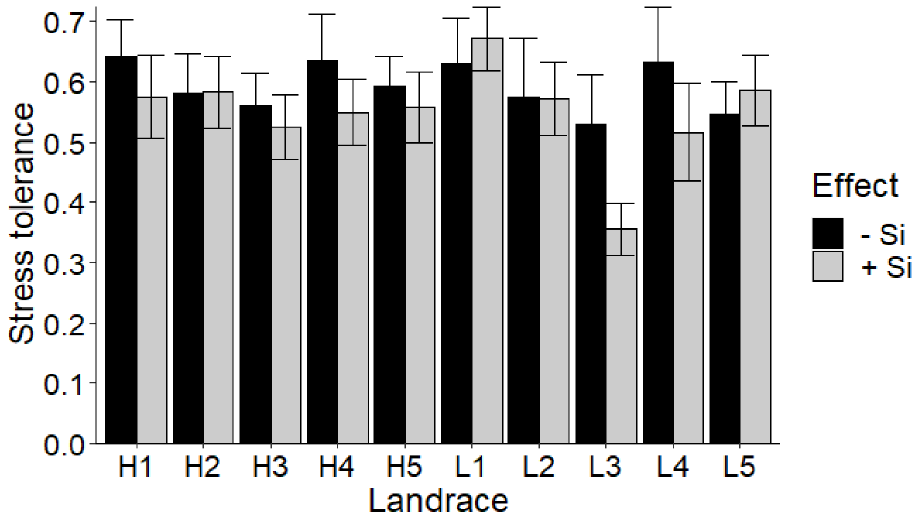

Figure S2: The impact of Si fertilisation on individual wheat landraces under drought stress. Stress tolerance was defined as shoot dry weight during drought stress divided by shoot dry weight in the control. Paired t-tests were used to compare the stress tolerance for – Si and + Si plants for each landrace. The effect of Si was not significant for any landrace ($p > 0.05$). Mean values ± SE are shown. N = 4.
